# Unraveling Reactivity Origin of Oxygen Reduction at High-Entropy Alloy Electrocatalysts with a Computational and Data-Driven Approach

**DOI:** 10.1021/acs.jpcc.4c01630

**Published:** 2024-06-29

**Authors:** Yang Huang, Shih-Han Wang, Xiangrui Wang, Noushin Omidvar, Luke E. K. Achenie, Sara E. Skrabalak, Hongliang Xin

**Affiliations:** † Department of Chemical Engineering, Virginia Polytechnic Institute and State University, Blacksburg, Virginia 24061, United States; ‡ Department of Chemistry, Indiana University - Bloomington, Bloomington, Indiana 47405, United States

## Abstract

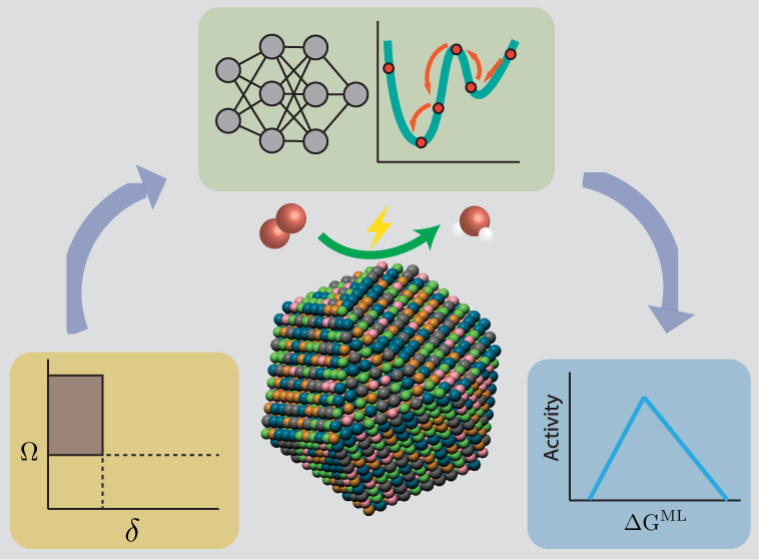

High-entropy alloys (HEAs), characterized as compositionally complex solid solutions with five or more metal elements, have emerged as a novel class of catalytic materials with unique attributes. Because of the remarkable diversity of multielement sites or site ensembles stabilized by configurational entropy, human exploration of the multidimensional design space of HEAs presents a formidable challenge, necessitating an efficient, computational and data-driven strategy over traditional trial-and-error experimentation or physics-based modeling. Leveraging deep learning interatomic potentials for large-scale molecular simulations and pretrained machine learning models of surface reactivity, our approach effectively rationalizes the enhanced activity of a previously synthesized PdCuPtNiCo HEA nanoparticle system for electrochemical oxygen reduction, as corroborated by experimental observations. We contend that this framework deepens our fundamental understanding of the surface reactivity of high-entropy materials and fosters the accelerated development and synthesis of monodisperse HEA nanoparticles as a versatile material platform for catalyzing sustainable chemical and energy transformations.

## 1 Introduction

The escalating issues of the energy crisis and environmental pollution underscore the urgency of the transition to a sustainable global economy. A pivotal aspect of this shift is the advancement of renewable energy technologies, especially those facilitating the conversion of chemical energy to electrical energy in fuel cells. At the heart of these energy conversion devices is the oxygen reduction reaction (ORR), whose efficiency is considerably hindered by the dearth of effective electrocatalysts.^1,2^ Platinum (Pt) stands as the most effective pure metal catalyst for the ORR, yet its widespread application is constrained by its high cost and scarcity. Efforts to reduce the loading of precious metals while enhancing site-specific activity have concentrated on the modification of Pt sites with strategies such as defect engineering, core–shell nanostructuring, and near-surface alloying.^3^ A major limitation of these Pt-based site ensembles is long-term durability, severely compromised by interfacial processes such as leaching, dealloying, and degradation dynamically occurring under operating conditions.^4^

High-entropy alloys (HEAs) represent a novel class of materials in a solid solution phase with five or more principal components and have demonstrated promising attributes across numerous application domains, including heterogeneous catalysis.^5−7^ Characterized by high configurational entropy, HEAs exhibit superior thermodynamic stability and miscibility beyond conventional alloys.^5^ Moreover, the exceptional diversity of multielement sites at HEA surfaces potentially introduces active site ensembles tailored for specific rate-determining steps. For instance, Pedersen et al. highlighted that traversing from simple Pt alloys to HEAs broadens the distribution of the binding energies of key reaction intermediates (e.g., *OH), increasing the likelihood of achieving optimal binding properties that align with the activity volcanoes.^8^ Numerous experimental investigations have underscored the significant promise of HEAs as ORR electrocatalysts.^9−13^ With a low-temperature solution-based approach, He et al. synthesized a CrMnFeCoNi HEA that demonstrated an exceptional ORR efficiency, showcasing a half-wave potential of 0.78 V and an onset potential of 0.88 V vs RHE, rivaling that of commercial Pt/C catalysts.^14^ In another study, Li et al. crafted an AlCuNiPtMn HEA with a modest Pt content of approximately 20–30 at % while exhibiting improved durability and ORR activity in comparison to commercial Pt/C.^15^ Furthermore, Bueno et al. fabricated PdCuPtNiCo HEA nanoparticles by annealing colloidally derived core@shell nanoparticles and showed them to be not only durable but also more active than commercial Pt catalysts for the ORR.^16^ The partial substitution of Pt/Pd with more Earth-abundant elements such as Ni, Co, and Cu drastically reduces the cost of electrocatalysts. Nonetheless, the underlying mechanisms of augmented activity have yet to be fully elucidated.

Despite the promising prospects of HEA catalysts, accurately predicting their stability and activity is challenging. The complexity of many-body interactions in HEAs precludes the development of accurate classical force fields, necessitating quantum mechanical treatments, which are impractical for large HEA nanoparticles with more than a few hundred atoms. Even with slab-model practice, the extraordinary diversity of local site environments on HEA surfaces renders high-throughput first-principles calculations of reactivity properties for each site infeasible. Although computational workflows accelerated by machine learning (ML) algorithms have been devised to model HEA catalysis,^8,17,18^ surface segregation—a critical phenomenon in the equilibrium configuration of alloys driven by symmetry breaking or reactive species—has been largely overlooked.

**Figure 1:**
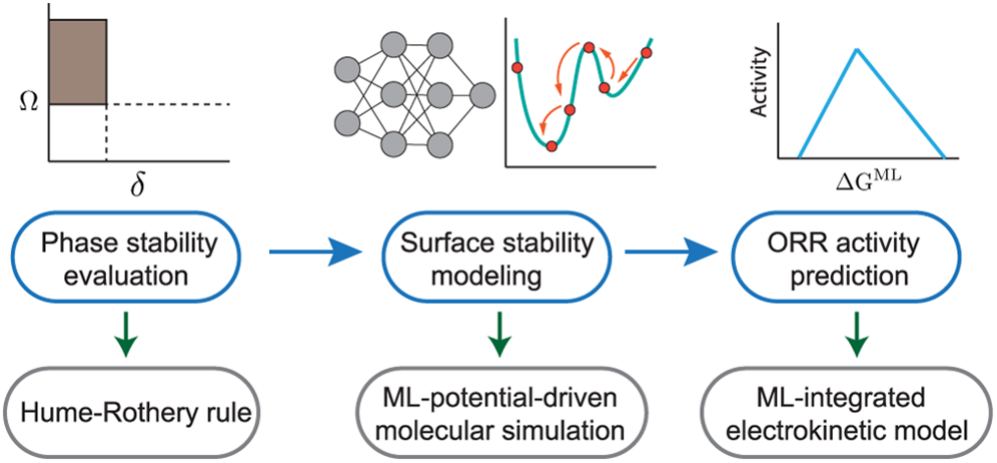
A computational and data-driven framework for the modeling of HEA catalysis.

In this work, we introduce a computational and data-driven framework to overcoming the aforementioned challenges, with our recently reported PdCuPtNiCo HEAs as a specific example.^16^ The observed discrepancy between experimentally measured ORR activity trends and those predicted by conventional modeling approaches, such as homogeneous mixing models, underscores the limitations of these methods in accurately capturing the complex heterogeneous elemental distributions within HEA nanoparticles. These models fail to account for the intraparticle heterogeneity and the resultant distribution of active sites, which could significantly influence the catalytic activity of HEAs under relevant conditions. As depicted in Figure 1, our framework encompasses three main components: phase stability evaluation using empirical thermodynamic rules, surface modeling of HEA nanoparticles through large-scale Monte Carlo simulations driven by deep learning interatomic potentials, and activity prediction via the integration of a pretrained theory-infused neural network (TinNet)^19^ that accurately predicts the surface reactivity of metal sites with a descriptor-based electrokinetic model. This framework successfully captures the enhanced ORR activity of the PdCuPtNiCo HEA nanoparticle system compared to pure Pt as observed in our experiment and elucidates the reactivity origin of HEA surface sites within the framework of the *d*-band theory. The framework not only facilitates accurate modeling of HEA catalysis but also advances our fundamental understanding of high-entropy materials’ surface reactivity, potentially accelerating the design and synthesis of monodisperse HEA nanoparticle catalysts for sustainable chemical and energy transformations.

## 2 Computational Methods

Spin-polarized density functional theory (DFT) calculations for all high-entropy alloy (HEA) systems were conducted using the Vienna Ab initio Simulation Package (VASP).^20,21^ The projector-augmented wave method was used to describe the electron–core interaction with a kinetic energy cutoff of 500 eV. The exchange-correlation energy was approximated through the generalized gradient approximation (GGA), adopting the revised Perdew–Burke–Ernzerhof (RPBE) functional.^22^ In preparation for the training of the deep learning interatomic potential, 120,085 structures were generated via the special quasi-random structure approach.^23^ This process ensured a comprehensive sampling within the configurational space, incorporating both bulk in the fcc-phase and surface slab structures with {111} orientation across diverse unit cells (e.g., 3 × 3 × 4, 3 × 3 × 5, 3 × 4 × 5, , ) and compositions (ranging from mono- to quinary-component systems with varied concentrations). Each slab configuration includes a 15 Å vacuum spacing to eliminate interactions between periodic images along the *z*-axis. The lattice constant of the Pd_0.25_Cu_0.20_Pt_0.20_Ni_0.22_Co_0.13_ HEA system of interest, 3.74 Å, was estimated based on the bulk composition using Vegard’s law.^24^ Considering the secondary effect of geometric relaxation on the energy trend in Monte Carlo simulations, only single-point calculations were executed.^25^ A Monkhorst–Pack mesh of 3 × 3 × 3 and 3 × 3 × 1 *k*-points was used to sample the Brillouin zone for bulk structures and surface slabs, respectively. The Methfessel–Paxton smearing scheme was used with a smearing parameter of 0.1 eV. Electronic energies were extrapolated to *k*_B_*T* = 0 eV.

The atomistic line graph neural network (ALIGNN),^26^ a flavor of message passing graph neural networks, was employed to predict the potential energy of the HEA systems. To train the ALIGNN models, the whole data set was randomly divided into training and test sets with the split ratio of 9:1. The trained ALIGNN models achieve a mean absolute error (MAE) of 2.664 meV/atom on the test data set. The detailed parameters of the ALIGNN models, such as the number and size of layers, optimizer, and learning rate, can be found in the Data/Code Availability section. The canonical MC simulations (NVT) were conducted with the trained deep learning potential to attain equilibrium configurations.^25^ We used the slab model of fcc(111) with a 10 × 10 × 50 lattice as a representative model of spherical nanoparticles. The thickness of this slab is approximately 10 nm, which closely matches the nanoparticle diameter in experiments.^16^ In the synthesis procedure,^16^ HEAs are annealed under a H_2_ gas environment at 900 K. For simplicity, H_2_ was not incorporated in our simulations because the hydrogen binding strengths are similar across transition metals considered here and the *H coverage is expected to be low at the annealing temperature of 900 K. In each MC step, two atoms are randomly selected and then swapped based on the acceptance probability min(1, e^–Δ^*^E^*^/^*^RT^*) where Δ*E* is the potential energy difference between the new and old configurations. We run 10 independent MC simulations with different randomly sampled initial configurations and obtain the segregation profile by taking the average and standard deviations on the 10 equilibrium surface configurations.

The pretrained theory-infused neural network (TinNet) without fine-tuning was used to predict OH adsorption energies.^19^ The models were trained on ∼1000 OH adsorption energies on the top site of fcc(111) metal and bimetallic alloy surfaces. The OH adsorption energy difference between HEA and Pt surface under the catalytically relevant OH coverage, 1/4, is calculated using surface slabs with 1/9 OH coverage as follows:
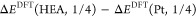
1
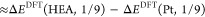
2
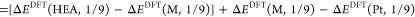
3
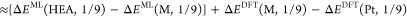
4where M refers to the pure metal M surface site with the same element as the adsorption site atom of HEA. The first approximation is made based on the fact that the effect of adsorbate coverage on adsorption energy usually depends on adsorbate–adsorbate interactions rather than on the surface itself. The 1/9 OH coverage on a HEA corresponds to one OH molecule being adsorbed on a 3 × 3 periodic unit cell which better captures the local environment compared to a 2 × 2 unit cell. On top of the TinNet-predicted OH adsorption energies, zero-point energy corrections and entropic contributions to the free energies were added for electrokinetic models, and the values were taken from ref^3^. The linear scaling relationships are employed in order to represent the free adsorption energy of each ORR intermediate using that of *OH on the same surface site. Specifically,  eV, .^2^

## 3 Results and Discussion

### 3.1 Phase Stability

**Figure 2:**
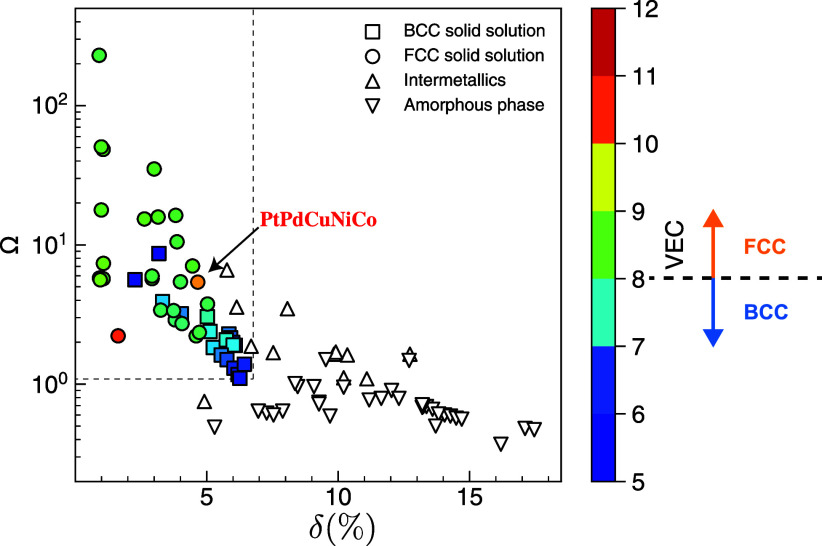
Phase stability is characterized by two criteria of Ω and δ. Each point represents a multiprincipal-component alloy or compound. The color coding is based on the valence electron concentrationn (VEC), which distinguishes between fcc and bcc solid solutions.

To ascertain the phase stability of a HEA system, we utilized the Hume–Rothery rules.^27^ These foundational principles elucidate the conditions under which a multiprincipal-component metallic system can form a solid solution or other phases. To predict the formation of a single-phase solid solution, two primary criteria have been widely used. The first is the atomic radius difference, quantifying the variation in atomic sizes of the components in a mixture or compound,
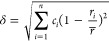
5where *c*_*i*_ represents the molar ratio of component *i* and *r* is the atomic radius. A higher value of δ indicates a greater disparity in the sizes of the atoms present, which can influence the material’s structural and electronic properties. A smaller δ is preferable to minimize internal stress, which impedes random diffusion and mixing. The second criterion is the Ω parameter,
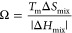
6representing the entropy driving force relative to the absolute magnitude of the mixing enthalpy. The enthalpy of mixing is calculated as
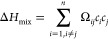
7where  is the regular solution interaction parameter between the *i*^*th*^ and *j*^*th*^ elements and  is the enthalpy of mixing of binary liquid alloys calculated based on the Miedema macroscopic model.^28^ A higher Omega value is essential to lower the Gibbs free energy, thus favoring the mixing process. Illustrated in Figure 2 is the schematic decision boundary described by δ and Ω that maps numerous multiprincipal-component metallic systems with their respective phases collected from ref (27). The dashed rectangle in the top-left signifies the domain of single-phase solid solutions. Moving beyond this domain results in the emergence of intermetallic compounds and amorphous structures. Additionally, the valence electron concentration (VEC, defined as the average number of valence electrons per atom) rule suggests that fcc solid solutions typically exhibit a higher VEC compared to bcc solid solutions.^29^ The VEC of our HEA system is 10 which is higher than the fcc/bcc VEC boundary located at around 8. As verified by experimental characterization, the Pd_0.25_Cu_0.20_Pt_0.20_Ni_0.22_Co_0.13_ HEA system of our interest forms a stable fcc single-phase solid solution, consistent with the decision boundaries in Figure 2.

### 3.2 Surface Stability

**Figure 3:**
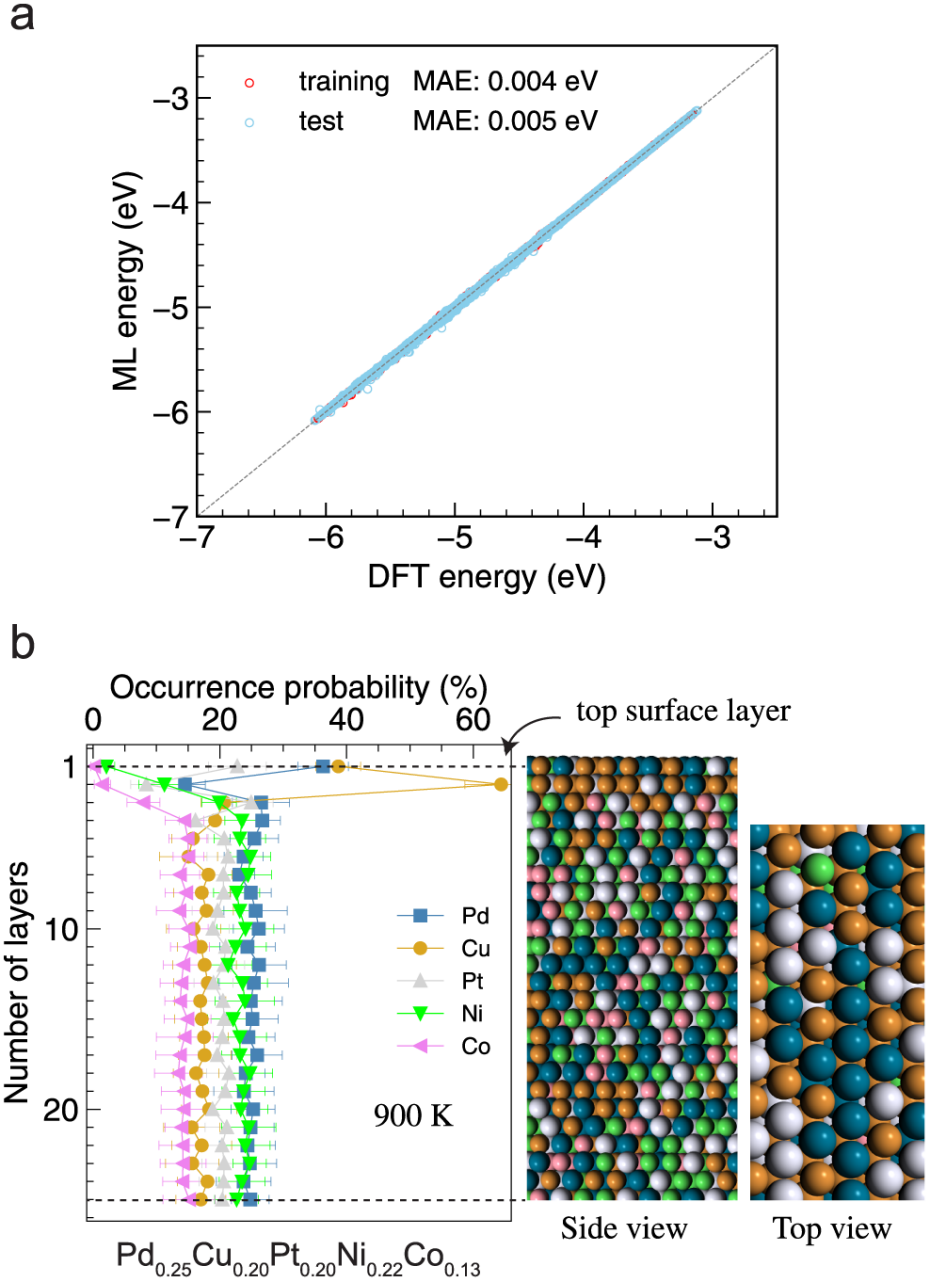
(a) The parity plot between DFT-calculated and ALIGNN-predicted energies per atom. (b) Surface segregation profile in the equilibrium configuration of the HEA.

To assess the surface stability of our HEA system under annealing conditions, we employed machine learning interatomic potentials based on graph neural networks, particularly the atomistic line graph neural network (ALIGNN), in molecular simulations to obtain equilibrium configurations. Details of model development can be found in the Method section. Figure 3a presents a parity plot comparing DFT-calculated and ALIGNN-predicted per-atom energies. Both training and test data sets exhibit mean absolute errors (MAEs) below 5 meV/atom, indicating a balanced model without noticeable underfitting or overfitting. To determine the equilibrium surface configurations after high-temperature annealing, canonical Monte Carlo simulations were conducted using the trained deep learning potential, with an equilibrium achieved after approximately 16,000 steps. The occurrence probability of each element across the surface layers, illustrated in Figure 3b, maps the segregation profile perpendicular to the surface. The average and standard deviation was obtained from 10 different initial samplings. This analysis revealed a pronounced segregation of Cu and Pd to the surface, with Pt only slightly higher than the bulk concentration, while Co and Ni were preferentially located in the bulk.

The underlying mechanisms driving Cu and Pd segregation can be elucidated through Helmholtz free energy components *F* = *U* – *TS* of canonical ensembles, encompassing internal energy (*U*) and entropy (*S*). According to the cohesion theory,^30^ the cohesive energy trend among late transition and noble metals is mainly dictated by the *d*-band, approximated as
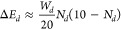
8where *W*_*d*_ represents the *d*-band width and *N*_*d*_ is the number of *d*-electrons. For Cu, with a fully occupied *d*-band (*N*^*d*^ = 10), its cohesive contribution is minimal, making metallic bonds with Cu energetically less favorable. Consequently, to minimize the system’s internal energy, Cu atoms tend to segregate onto the surface. Pd has only marginally higher cohesive and surface energies compared to Cu,^31^ which partially contributes to the surface segregation of Pd. Moreover, given the abundance of Pd in the HEA’s bulk composition (Pd_0.25_Cu_0.20_Pt_0.20_Ni_0.22_Co_0.13_ as targeted in our simulations), segregation to the surface occurs as the bulk strives for an equimolar distribution to maximize the configurational entropy,
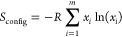
9thereby rationalizing the segregation of Pd atoms at the HEA surface as observed in Monte Carlo simulations. For the remaining metals—Co, Ni, and Pt—their cohesive energies and surface energies are all larger than those of Cu and Pd,^31^ which is why they do not exhibit surface segregation.

### 3.3 ORR Activity

**Figure 4:**
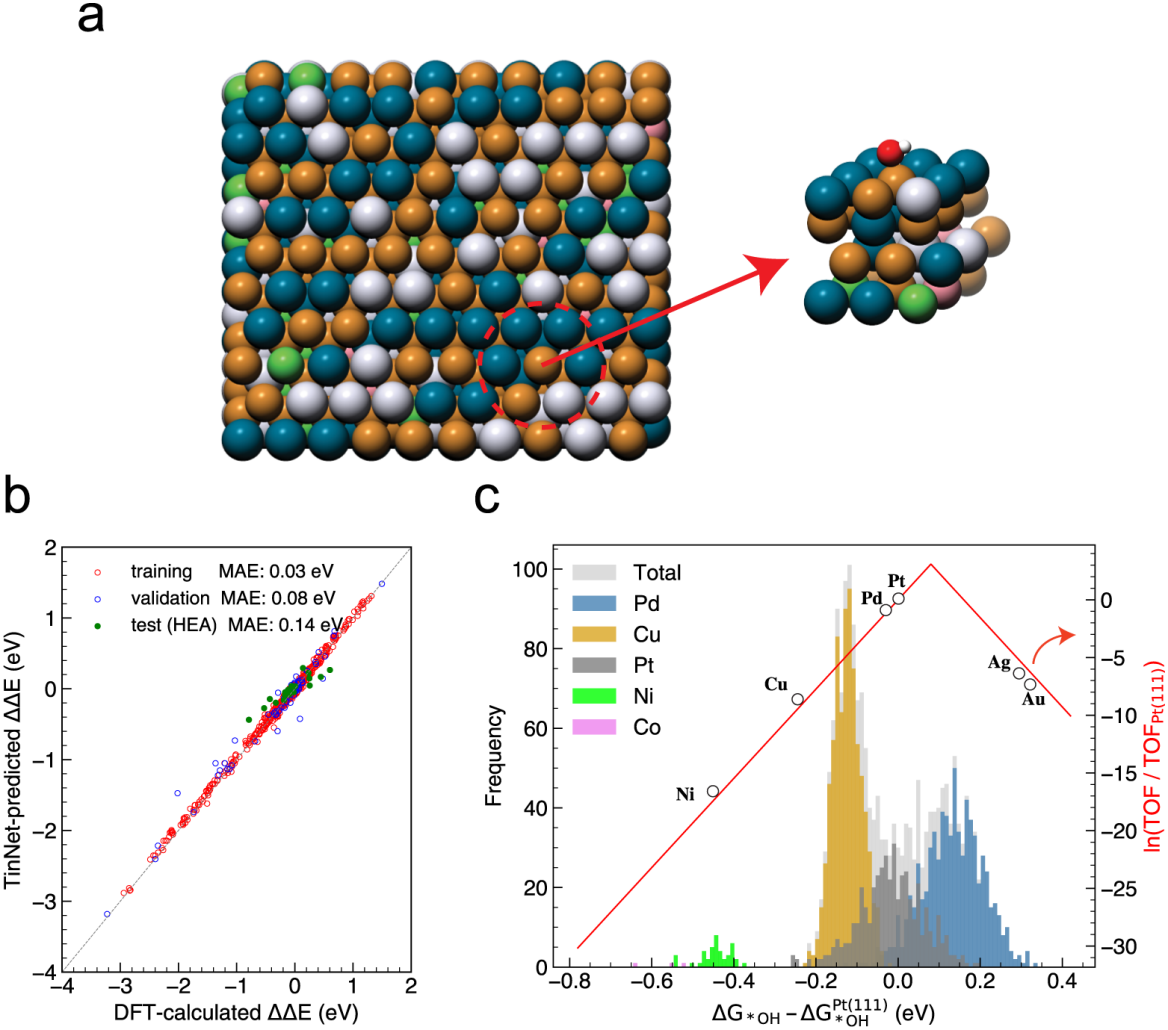
(a) Slab segmentation. (b) Parity plot between DFT-calculated and TinNet-predicted changes in OH adsorption energies. (c) ORR activity map and OH adsorption energy distribution histograms in the equilibrium configuration of HEA surfaces.

For equilibrium configurations from Monte Carlo simulations, we aimed to predict the ORR activity of surface sites. We used OH adsorption energies as the surface reactivity descriptor. As depicted in Figure 4a, the equilibrium configuration of a large slab (10 × 10 × 50) can be sampled with 3 × 3 × 4 sub-slabs while considering periodic boundary conditions. Each small slab’s central surface atom represents a surface site within the whole slab of 10 × 10 × 50. The 3 × 3 × 4 unit cell, encompassing all first-nearest neighboring atoms around the surface site, is sufficiently large to capture the local environment primarily determining the chemisorption properties of the surface site while being computationally feasible in DFT. For simplification, the OH adsorption energy at the atop site is designated as the reactivity descriptor, given its stability on the 111 facet of most transition and noble metals, particularly with the consideration of ice bilayer structures.^32−34^ To mitigate errors stemming from deviations in OH surface coverage under the reaction conditions, we focus on predicting changes in the intrinsic reactivity caused by variations in the local environment relative to pure metal (111) surface sites. The change in the reactivity descriptor can be accurately predicted using our pretrained TinNet model due to error cancellation.^35^ This precision is highlighted in the parity plot, which compares DFT-calculated adsorption energies against those predicted by TinNet, presented in Figure 4b. The plot demonstrates that a test mean absolute error (MAE) of 0.14 eV on HEAs, though higher than that of training set (0.03 eV) and validation set (0.08 eV) due to the complexity of the multielemental sites on HEAs, is relatively small considering the DFT uncertainty at a similar magnitude, underscoring the model’s efficacy in capturing the shifts in reactivity descriptors.

In the electrokinetic model to determine ORR activity,^36^ the four-electron transfer mechanism is adopted, with site-specific reaction rate quantified by the highest thermodynamic barrier among the four elementary electron-transfer steps, at equilibrium potential of 1.23 V vs RHE,
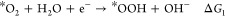
10
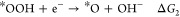
11
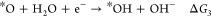
12
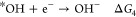
13
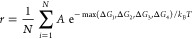
14where *N* denotes the number of surface sites and *A* is the pre-exponential factor which is assumed to be the same for all the surface sites. The ORR activity relative to Pt(111) is defined at the equilibrium potential as ln (*r*/*r*_Pt_). Employing linear scaling relationships between the adsorption energies of all ORR intermediates and OH yields a volcano-shaped activity function over OH adsorption free energy relative to Pt(111) (refer to Figure 4c). A histogram of the TinNet-predicted OH adsorption free energy difference, with surface sites categorized by elements, is plotted (Figure 4c). Notably, numerous sites align near the activity peak, resulting in the HEA’s specific activity being 2.16 times that of Pt(111). This enhancement in activity primarily stems from Pd (1.55 out of 2.16) and Pt (0.60 out of 2.16) sites, with Cu sites contributing minimally (0.01 out of 2.16), suggesting an optimization strategy that reduces Cu to increase the number of active surface sites. Interestingly, compared to pure metals, the distribution of Cu and Pd site reactivity exhibits a significant shift toward the weakening side, whereas Pt sites show no systematic shifts.

**Figure 5:**
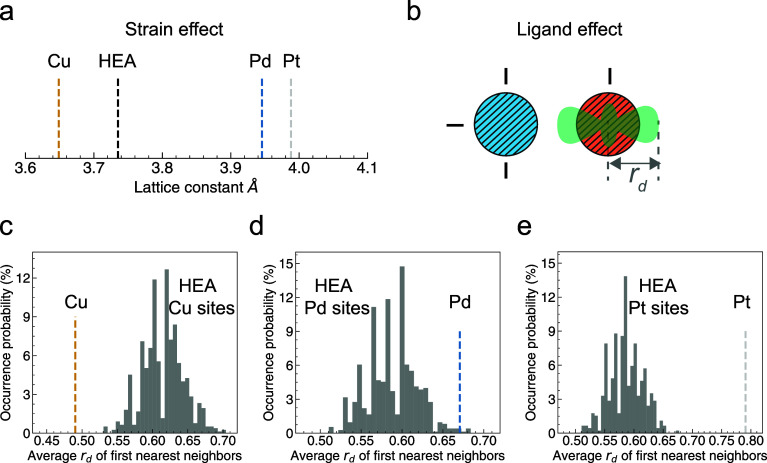
(a) The lattice constants of HEA, Cu, Pd, and Pt. (b) The ligand effect. The distribution histogram of average *r*_*d*_ of the first nearest neighbors of (c) Cu sites, (d) Pd sites, and (e) Pt sites on the surface of the equilibrium configuration of the HEA.

To unravel the reactivity origin of the surface sites, specifically, the *d*-band theory^37^ is employed for further analysis. In transition and noble metals, a low-lying *d*-band or *d*-band center tends to weaken orbital hybridization and, thus, OH adsorption on surface sites. There are two main factors of the *d*-band center shift, the strain effect characterized by the bulk lattice constant and the ligand effect governed by the *d*-orbital radius *r*_*d*_ of the neighboring atoms (see Figure 5b).^38^ A reduced lattice constant or enlarged *d*-orbital radius of the neighbors tends to increase *d*–*d* orbital coupling between the site atom and its neighbors, lowering the *d*-band center and weakening OH binding. The *r*_*d*_ values of Cu, Pd, Pt, Ni, and Co are 0.49, 0.67, 0.79, 0.52, and 0.56 Å, respectively. The distribution histogram of the average *r*_*d*_ of first-nearest neighbors of the three types of surface sites in HEAs are plotted in Figure 5c–e. For Cu sites in the HEA, the lattice expansion reative to the pure Cu (see Figure 5a) weakens the orbital coupling and strengthens OH binding. Due to a small *r*_*d*_ of Cu compared to its neighboring metals, the increase of interorbital coupling in HEAs weakens OH binding. If the ligand effect dominates, then OH binding on Cu sites shifts to a weakening side. For Pd sites, opposite to Cu sites, the lattice compression strengthens orbital coupling and weakens OH binding. However, due to a large *r*_*d*_ of Pd compared to its neighboring metals, the decrease of interorbital coupling in HEAs strengthens OH binding. We can postulate that the strain effect dominates in this scenario, which leads to a weakened OH binding distribution as observed in Figure 4c. For Pt sites, the two effects work in the different directions; as a result, no systematic shifts of OH binding were observed on Pt sites.

## 4 Conclusions

In conclusion, our study introduces an integrated computational and data-driven approach for understanding high-entropy alloy (HEA) catalysis. This framework has effectively demonstrated its capability by unraveling the origin of enhanced ORR activity of the PdCuPtNiCo HEA system, as previously observed in our experiment. Our approach stands as a significant step forward in multiscale simulations of HEAs in catalysis, offering physical insights into the surface behavior of high-entropy materials in catalytic reactions, including but not limited to ORR. Furthermore, the versatility and predictive power of this framework signal accelerating the development and optimization of monodisperse HEA nanoparticles for surface reactions, opening up a promising path for their applications in advancing sustainable energy solutions.

## Data Availability

Currently, all source data along with Jupyter Notebooks for data preprocessing, model development, and *post hoc* analysis are available from the GitHub repository: https://github.com/hlxin/orr-hea.
